# Collection and Visualization of Dietary Behavior and Reasons for Eating Using Twitter

**DOI:** 10.2196/jmir.2613

**Published:** 2013-06-24

**Authors:** Melanie Hingle, Donella Yoon, Joseph Fowler, Stephen Kobourov, Michael Lee Schneider, Daniel Falk, Randy Burd

**Affiliations:** ^1^University of ArizonaDepartment of Nutritional ScienceUniversity of ArizonaTucson, AZUnited States; ^2^University of ArizonaDepartment of Computer ScienceUniversity of ArizonaTucson, AZUnited States; ^3^Niagara Computer Services, Inc.Jenkintown, PAUnited States

**Keywords:** dietary behavior, data visualization, social media, mobile health, mHealth

## Abstract

**Background:**

Increasing an individual’s awareness and understanding of their dietary habits and reasons for eating may help facilitate positive dietary changes. Mobile technologies allow individuals to record diet-related behavior in real time from any location; however, the most popular software applications lack empirical evidence supporting their efficacy as health promotion tools.

**Objective:**

The purpose of this study was to test the feasibility and acceptability of a popular social media software application (Twitter) to capture young adults’ dietary behavior and reasons for eating. A secondary aim was to visualize data from Twitter using a novel analytic tool designed to help identify relationships among dietary behaviors, reasons for eating, and contextual factors.

**Methods:**

Participants were trained to record all food and beverages consumed over 3 consecutive days (2 weekdays and 1 weekend day) using their mobile device’s native Twitter application. A list of 24 hashtags (#) representing food groups and reasons for eating were provided to participants to guide reporting (eg, #protein, #mood). Participants were encouraged to annotate hashtags with contextual information using photos, text, and links. User experience was assessed through a combination of email reports of technical challenges and a 9-item exit survey. Participant data were captured from the public Twitter stream, and frequency of hashtag occurrence and co-occurrence were determined. Contextual data were further parsed and qualitatively analyzed. A frequency matrix was constructed to identify food and behavior hashtags that co-occurred. These relationships were visualized using GMap algorithmic mapping software.

**Results:**

A total of 50 adults completed the study. In all, 773 tweets including 2862 hashtags (1756 foods and 1106 reasons for eating) were reported. Frequently reported food groups were #grains (n=365 tweets), #dairy (n=221), and #protein (n=307). The most frequently cited reasons for eating were #social (activity) (n=122), #taste (n=146), and #convenience (n=173). Participants used a combination of study-provided hash tags and their own hash tags to describe behavior. Most rated Twitter as easy to use for the purpose of reporting diet-related behavior. “Maps” of hash tag occurrences and co-occurrences were developed that suggested time-varying diet and behavior patterns.

**Conclusions:**

Twitter combined with an analytical software tool provides a method for capturing real-time food consumption and diet-related behavior. Data visualization may provide a method to identify relationships between dietary and behavioral factors. These findings will inform the design of a study exploring the use of social media and data visualization to identify relationships between food consumption, reasons for engaging in specific food-related behaviors, relevant contextual factors, and weight and health statuses in diverse populations.

## Introduction

The high prevalence of obesity continues to be a major public health challenge in the United States [1], contributing to increased incidence of weight-related comorbidities (eg cancer, type 2 diabetes, cardiovascular disease) and early mortality [2]. The societal burden of obesity is substantial, including loss of productivity, increased health care costs, and decreased quality of life [3]. Collectively, these economic and social costs significantly threaten the stability of our society.

The dramatic increase in obesity over the past 3 decades is attributed to a mismatch between humans’ evolved physiology and the contemporary environment, characterized by an abundance of high-calorie foods with few opportunities for equivalent energy expenditure [4]. Within this obesogenic environment, individuals must exert a significant and sustained level of self-regulation to avoid weight gain.

It has been suggested that an overall increased availability of calories (and subsequent increased energy intake) can explain a significant proportion of the increase in obesity prevalence at the population level [5]. Data from nationally representative diet and health behavior surveys (Nationwide Food Consumption Survey, 1977-78; the Continuing Survey of Food Intakes of Individuals, 1989-91; and the National Health and Nutrition Examination Surveys, 1994-98 and 2003-06) suggest that increases in energy intake over the past 30 years may be attributed to an increased frequency of eating and drinking occasions (in particular, snacking occasions) and larger portion sizes [5]. These 2 dietary behaviors represent specific and modifiable intervention targets, whereby excess energy intake may be reduced and excess weight gain prevented if strategic reductions are achieved.

Despite extensive evidence-based dietary guidance directed at consumers (eg, Healthy People 2010 and the Dietary Guidelines [6,7]), implementation of healthy lifestyle habits by Americans remains lacking [8]. Nutrition education research has established that knowledge about healthy eating is necessary but not sufficient to change behavior [9]. Qualitative and quantitative studies of eating behavior have confirmed that decisions around eating behavior are often unconscious and based on established habits [10]. As a result, dietary choices are often reactively determined by where, when, and with whom an individual eats rather than a product of conscious planning. Given the disproportionate availability of unhealthy compared to healthy foods, there is a high probability that the most readily available foods are not low calorie or nutrient-dense choices.

In the midst of this Information Age [11], the rapid advent of wireless technologies has already begun to revolutionize personal health. The number of smartphone users is increasing and wireless devices are rapidly expected to become the number one way by which consumers will access information and resources on the World Wide Web [11]. This rise of mobile health devices and software applications is projected to continue well into the next 5 years, wherein over 80% of wireless device purchases in 2016 will be for systems to monitor and improve health [12]. The ubiquity of mobile device ownership [13] has placed the power to influence health directly into the hands of consumers, including the ability to record and access personalized health information continuously, in real time, and from any location. At the same time, ready access to the large amounts of data collected through mobile devices and applications remains one of the greatest challenges facing the utility of mobile health (mHealth). The use of mobile devices and the resultant big data to influence health behavior change can only be realized if the data are translated into easily understood and actionable steps in real life. Mobile interfaces which provide users with access to their own data in ways that will help them to make optimal choices for their age, life stage, location, culture, beliefs, and resources is a critical—and often overlooked—aspect of existing mHealth applications.

Despite a large number of software applications available in mobile devices (iOS, Android, Microsoft, and Blackberry) to help individuals track eating behavior and make healthy food choices, most lack empirical data supporting their utility as health promotion tools. In addition, commercial applications often use proprietary methods of calculating energy intake/expenditure or restrict the type and amount of data that are collected and provided to the individual user which may obscure critical information from the user and perpetuate misinformation about dietary behaviors, nutrition, and health.

Changing a pattern of established behavior—particularly diet—is a complex process, and barriers to engaging in and maintaining dietary behavior change are well documented [14]. Extensive research suggests that eliminating an undesirable behavior does not involve unlearning, but rather *new* learning, and the physical environment and social context in which learning takes place (eg, emotions, time) will influence the learning process [15]. Thus, strategies designed to help individuals engage in and maintain changes in dietary behavior should acknowledge and address this complexity, providing critical information and feedback when most needed, including when, where, and with whom food choices and eating behaviors are occurring. A first step toward understanding the complexity of eating behavior is developing and testing a method to capture these critical data.

Therefore, the purpose of this study was to test the feasibility and acceptability (including usability) of a popular and free social media application (Twitter) to capture food and beverage consumption behavior and reasons for eating over 3 days in a group of young adults. A secondary aim was to capture and analyze user data from Twitter using a novel analytic tool, and visualize these data using behavioral maps to identify patterns in intake and behavior, and potential relationships between foods and contextual factors.

We posited that by tracking typical dietary choices and reasons for making food-related decisions simultaneously and in real time, relationships between these factors would emerge, and the addition of contextual factors, such as when, where, and with whom a person eats, would enrich these data providing additional information related to diet quality and potential mediators of dietary behavior.

## Methods

### Design, Participants, Recruitment, and Setting

Fifty adults were recruited from the University of Arizona between February and April 2012. Recruitment activities consisted of email advertisements sent to undergraduate listservs and announcements in undergraduate and graduate student classes. Inclusion criteria for the study included ownership of a Web-enabled mobile phone or smartphone, willingness to use a study-designated Twitter account for all study activities, and age older than 18 years at time of enrollment.

Respondents were invited to attend information sessions at the University of Arizona’s Department of Nutritional Sciences and informed consent was obtained from those who were interested and eligible. Participants were provided instructions on how to access Twitter and activate a study Twitter account using a nonidentifiable study-assigned Twitter handle, and assigned a study orientation date.

### Procedures

During the 1-hour study orientation session, participants were trained to use Twitter to record all food and beverages consumed over 2 weekdays and 1 weekend day, and their reasons for choosing these foods and beverages. Participants were asked to report these data using hashtags (#), Twitter’s electronic shorthand that is used to describe people, places, and things in a consistent manner. A list of 24 hashtags plus examples were provided to guide reporting and categorization of foods and reasons for eating. The list was informed by nomenclature used to describe food groups in nutrition education materials developed by the US Department of Agriculture (ie, the ChooseMyPlate) and reasons for eating commonly reported in the behavioral literature [16]. Additionally, participants were instructed to annotate hashtags with descriptive information (eg, short phrases, photos, or links) to provide additional information about where, when, why, and with whom specific diet behaviors occurred.

Following the orientation session, participants recorded their diet and reasons for eating for 3 consecutive days using their study-provided username and password and their mobile phone’s native Twitter application. All subsequent Twitter messages (tweets) were visible on the public Twitter stream, but authors were not identifiable.

The usability and acceptability of this approach was evaluated using a comprehensive evaluation approach that involved 2 complementary methods [17], real-time feedback from participants who were encouraged to email the study coordinator with technical problems or questions during the 3 days, combined with a 9-item exit survey that evaluated acceptability (satisfaction) of the experience after completion of data collection (see Multimedia Appendix 1). Survey responses were collected using SurveyMonkey [7], a commercially available Web-based service. Questions included participant preferences regarding Twitter access (ie, mobile platform vs desktop interface) and an overall rating of the application’s usability (eg, how easy/difficult it was to record their food intake using the application). Participants were also asked whether they felt adequately trained to use the application within the context of the study, and how—if at all—they would like to see the results of their tracking displayed. Participants were provided US $20 cash incentive upon completion of the exit survey. Permission to conduct the study was obtained from the Institutional Review Board at the University of Arizona.

### Data Analysis

Participant data were downloaded from the public Twitter stream using a novel Web-based data capture application (ViBE), developed specifically to identify and retrieve data from Twitter. For each participant, ViBE automatically calculates the frequency of hashtag occurrences in the Twitter stream during the designated study period, as well as the frequency of co-occurrences with other hashtags. Occurrence and co-occurrence data were merged and compiled as a normalized matrix. The matrix was exported from ViBE and visually processed using the algorithmic framework of GMap 1988 version 2.29.0 (AT&T Research) as a complete edge-weighted graph in which the Twitter hashtags and their matrix values correspond to the vertices and edge weights of the graph, respectively (see Multimedia Appendix 2). Co-occurrences are displayed on the resulting graph or map as adjoining “countries.”

The canonical map was produced by GMap [17,18], included with the Graphviz [19,20] graph visualization software (AT&T Research), which is an intuitive visualization of the relationships between patterns of foods and beverages consumed, and diet-related behaviors. GMap maps have been used for a variety of behavioral applications, including understanding purchasing of Amazon.com consumers [17]. Using these maps, users are able to see behavioral patterns and infer relationships. GMap has been further augmented by AT&T Research to handle streaming feeds from Twitter [21] and addresses the challenge of real-time data collection and visualization [22].

## Results

### Feasibility

**Figure 1:**
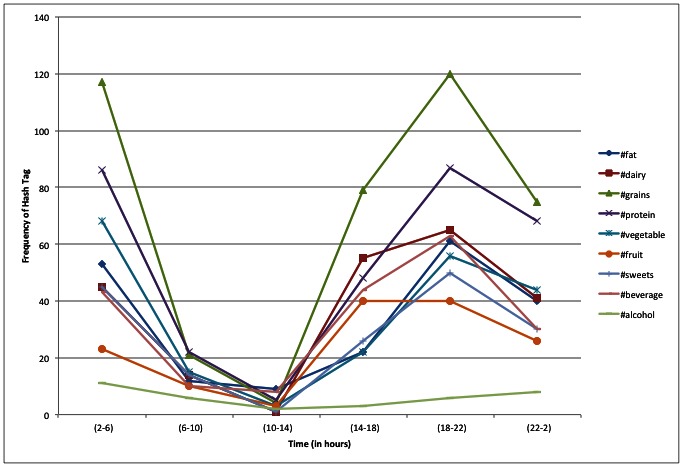
Frequency and timing of food hashtags over a 24-hour time period.

**Figure 2:**
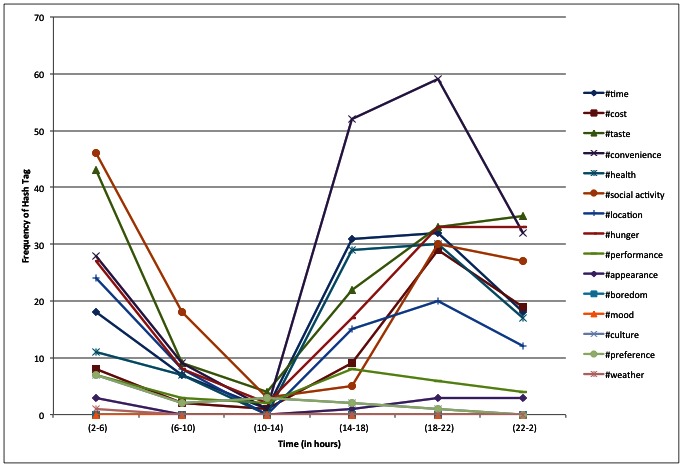
Frequency and timing of behavior hashtags over a 24-hour time period.

All participants were able to use Twitter and the study-provided list of hashtags to report their food and beverage intake and related dietary behaviors for 3 consecutive days. A total of 773 tweets containing 2862 hashtags were reported (1756 food-related hashtags and 1106 reasons for eating hashtags). The 3 most frequently reported food-related hashtags were #grains (21%), #protein (17%), and #dairy (13%), whereas the most frequently reported reasons for eating were #convenience (16%), #taste (13%), and #social (11%) (Figures 1 and 2). A total of 164 errors in hashtag spelling were observed (141 food-related hashtags and 23 behavioral hashtags were misspelled). In addition, participants also created 30 of their own hashtags (which were reported 40 times during the course of the study; see Table 1).

### Data Visualization

**Figure 3:**
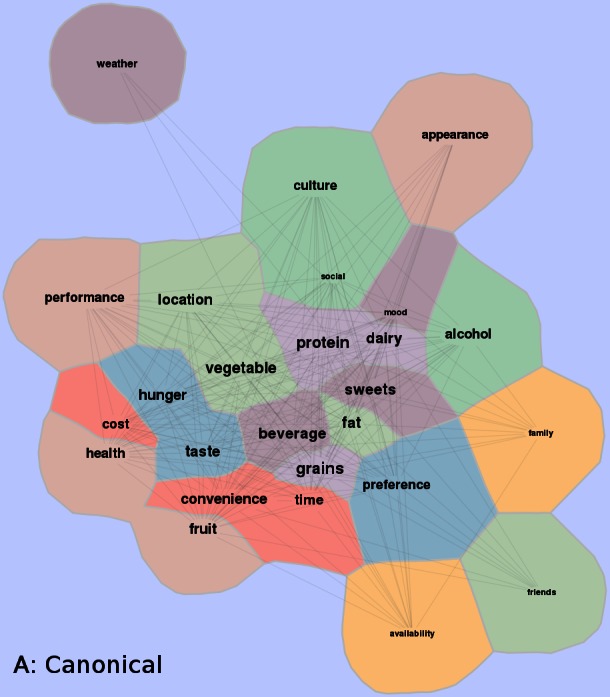
Study-generated data collected were transformed into a co-occurrence matrix and then applied to a visually representative map. Common co-occurrence hashtags placed in a country denoted by different colors on the map. Frequency of hashtags is also shown with centrally located countries contributing to higher frequencies and peripheral countries contributing to lower frequencies of hashtags.

**Figure 4:**
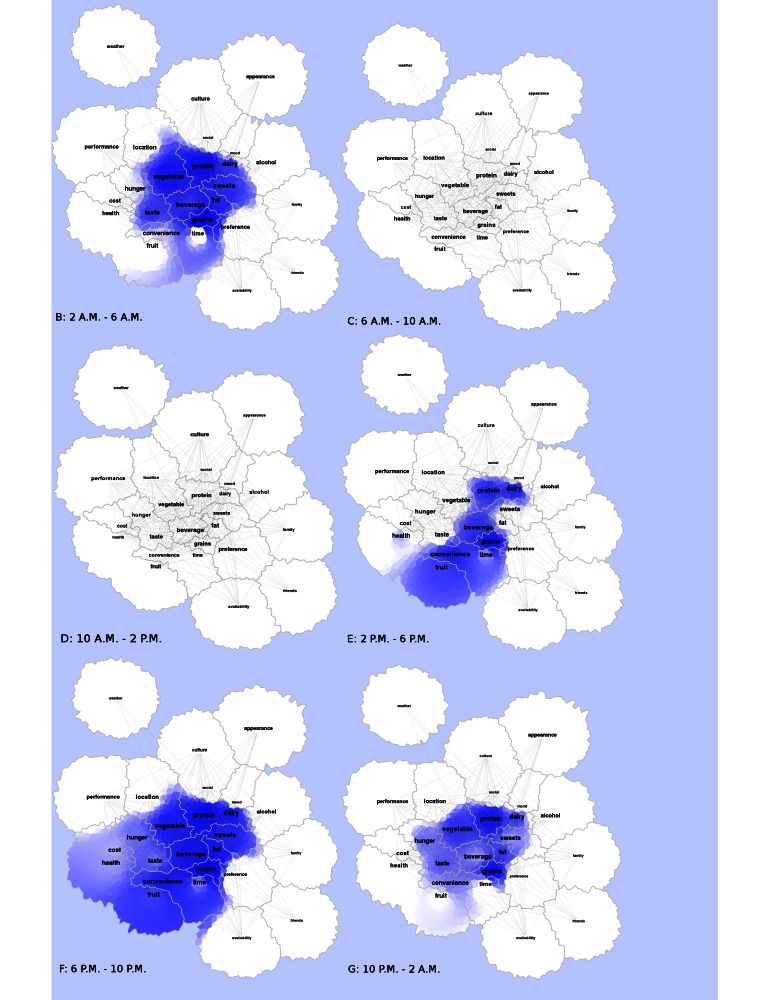
Heat maps showing frequencies throughout a 24-hour time period for study-generated data are shown. Higher frequencies are displayed with a darker blue hue, whereas progressively lower frequencies are lighter in blue. White coloration refers to little to no frequency activity.

**Figure 5:**
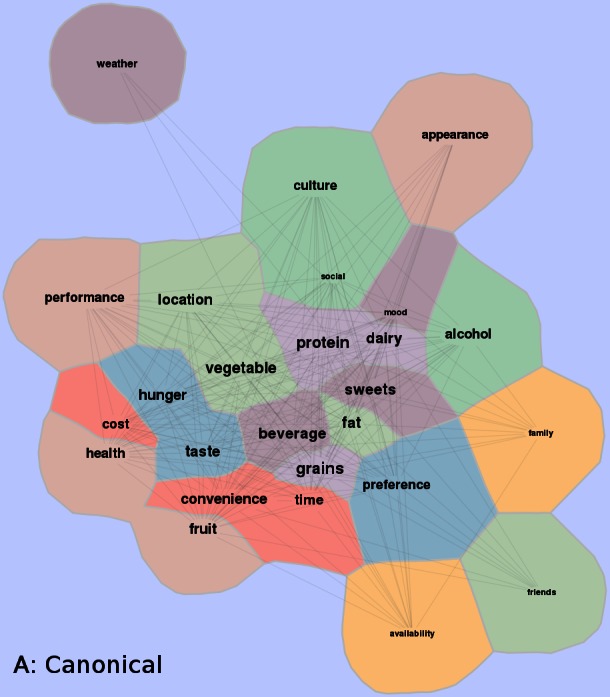
Study-generated hash-tags over a 24-hour time period. Study-generated data collected were transformed into a co-occurrence matrix and then applied to a visually representative map. Common co-occurrence hashtags placed in a country denoted by different colors on the map. Frequency of hashtags is also shown with centrally located countries contributing to higher frequencies and peripheral countries contributing to lower frequencies of hashtags.

**Figure 6:**
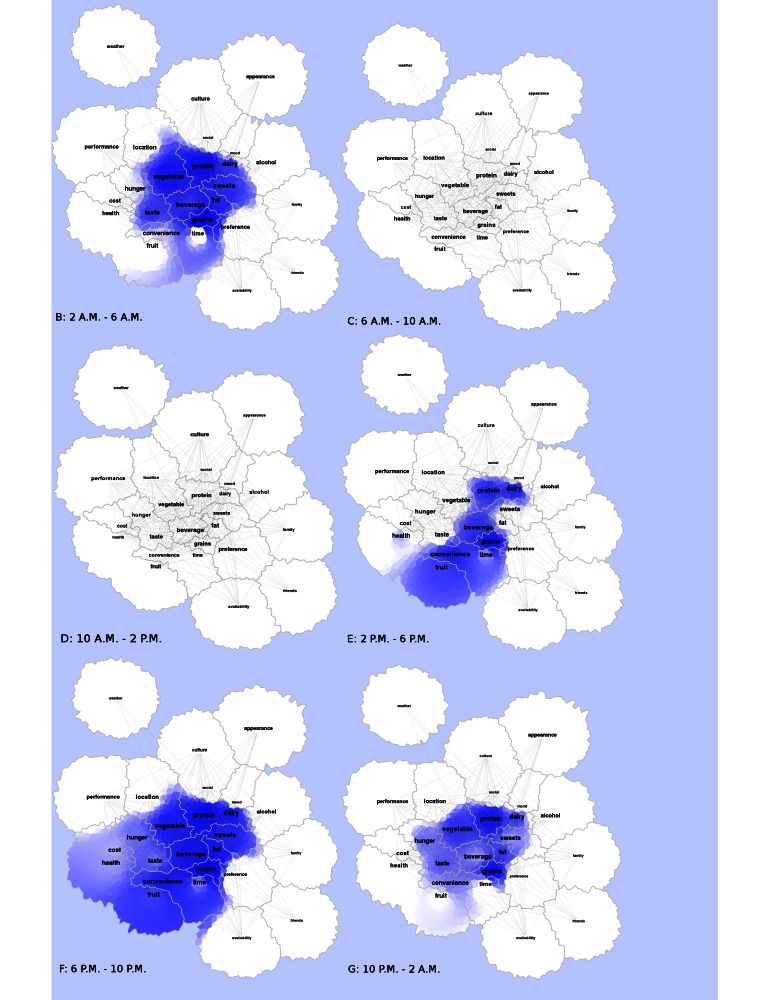
Study-generated hashtags over a 24-hour time period. Heat maps showing frequencies throughout a 24-hour time period for study-generated data are shown. Higher frequencies are displayed with a darker blue hue, whereas progressively lower frequencies are lighter in blue. White coloration refers to little to no frequency activity.

The ViBE software was used to retrieve data from Twitter and determine the frequency of occurrences and co-occurrences. This matrix was imported into the GMap software platform, and several canonical maps (Figures 3 and 5) were produced. The most frequently co-occurring hash tags were clustered into “countries” designated by a unique color. The data were separated into 4-hour time increments (6 am-10 am, 10 am-2 pm, 2 pm-6 pm, 6 pm-10 pm, 10 pm-2 am, and 2 am-6 am), and frequency of hashtag occurrence by time of day was explored over 24 hours. Frequency of hash tag reporting during 4-hour time blocks are represented by the intensity of color, in which darker colors represent more frequent hashtag reporting (Figures 4 and 6). The most active reporting periods were observed between 2 pm and 6 am (92% of all tweets).

### Acceptability

During the 3-day data collection period, 3 of 50 participants contacted the study coordinator via email and Twitter to receive help on classifying specific foods (2 questions), and constructing a hashtag that involved spaces (1 question). Additional survey data was collected from participants upon completion that suggested the Twitter platform was well received by participants overall. There was ambiguity regarding the use of several hash tags (eg, #appearance, which participants interpreted as either a person’s appearance or the food’s appearance). Participants also suggested that the ability to add their own hashtags should be an option to more precisely describe food choices and reasons for eating.

Of the 50 participants, 56% used only their mobile phones to access Twitter, 8% used their desktop or laptop computer, and 36% used a combination of mobile phone and desktop or laptop computer. Most participants who used both methods preferred the mobile phone to the computer. Although 73% (38/50) rated Twitter as very easy to use, 10% (5/50) reported that the 140-character limit imposed by the application made it a challenge to accurately report foods and reasons for eating. Many participants 36% (8/50) described the use of Twitter to record diet and behavior as a positive experience. Six participants (12%) recommended increasing the study duration to obtain a more comprehensive summary of their food intake and related dietary behavior, and 18 participants (36%) also commented about providing feedback to users about diet quality and suggestions on how to improve diet quality.

**Table 1:** Frequency of hashtags reported by participants (50 participants over 3 consecutive days).

Hashtags			Frequency
**Study-provided**			

	**Food-related**		
		#alcohol	41
		#beverage	198
		#dairy	221
		#fat	174
		#fruit	131
		#grains	365
		#protein	307
		#sweets	150
		#vegetable	169

	**Behavior-related**		
		#appearance	5
		#boredom	0
		#convenience	173
		#cost	68
		#culture	14
		#health	94
		#hunger	120
		#location	79
		#mood	103
		#performance	30
		#preference	41
		#social	122
		#taste	146
		#time	109
		#weather	2

**Participant-generated**			
	**Food-related**		
		#bread	1
		#butter	1
		#carbohydrates	2
		#carbs	4
		#coffee	1
		#lotsoffat	1
		#meat	4
		#meats	1

	**Behavior-related**		
		#2	1
		#3-	1
		#aapl	1
		#breakfast	1
		#comfort	1
		#craving	2
		#easy	1
		#feed	1
		#friend	1
		#goingtofeelitinthemorning	1
		#habit	2
		#happy	1
		#healthy	2
		#home	1
		#leftovers	1
		#lunch	1
		#price	1
		#random	1
		#still	1
		#test	1
		#timeegg	1
		#vibestudy	1

**Figure 7:**
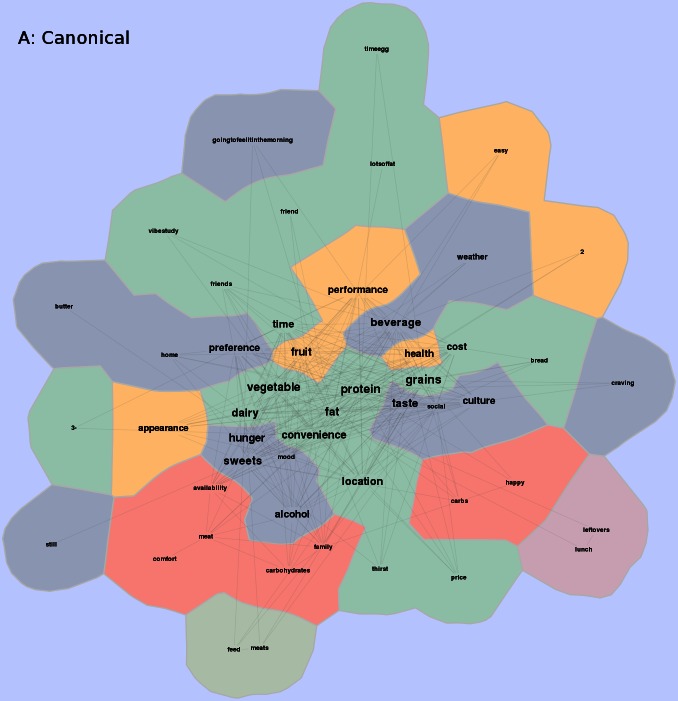
Study-generated and participant-provided hash tags over a 24-hour time period. Study-generated and participant-provided data collected were transformed into a co-occurrence matrix and then applied to a visually representative map. Common co-occurrence hashtags placed in a country denoted by different colors on the map. Frequency of hashtags is also shown with centrally located countries contributing to higher frequencies and peripheral countries contributing to lower frequencies of hashtags.

**Figure 8:**
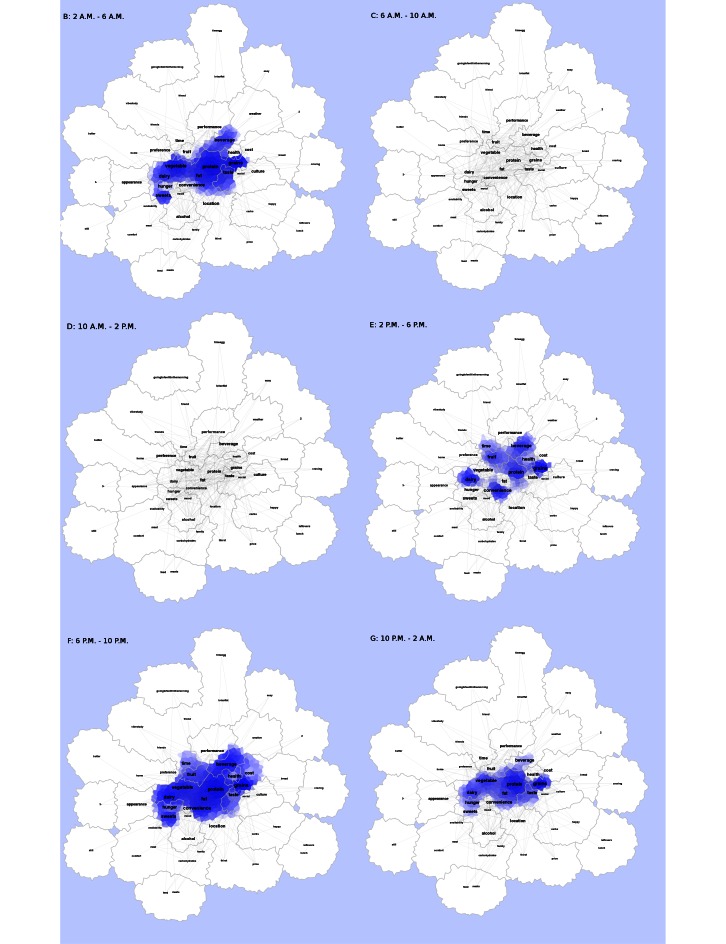
Study-generated and participant-provided hash tags over a 24-hour time period. Heat maps showing frequencies throughout a 24-hour time period are shown for study-generated and participant-provided data. Higher frequencies are displayed with a darker blue hue, whereas progressively lower frequencies are lighter in blue. White coloration refers to little to no frequency activity.

## Discussion

### Principal Findings

This study demonstrates that Twitter is a feasible and acceptable method for capturing real-time dietary intake and behavior, and that the resulting data can be used to visualize relationships between food consumption and reasons for engaging in these behaviors. This approach represents a novel method for collecting and characterizing dietary and behavioral data. In addition, this study demonstrates the capacity to visualize dietary behaviors using maps as well as adding valuable contextual dimensions, such as time of day and location. Identification of patterns of behavior and relationships between behaviors and time are important factors in understanding and creating strategies to influence established patterns of behavior, such as diet.

In the future, additional parameters could be integrated into heat maps to further enhance the visualizations, eg, phenotype, genotype, or sociodemographic data. Further illustrating the defining characteristics of a population will increase understanding of relationships between nutrition and health and disease states.

Our approach has utility for health interventions. Using these data collection and visualization techniques, data from individuals could be co-located on a map with others, leading to identification of common characteristics. As an example, tweets from a specified body mass index (BMI), such as those with a BMI greater than 25 could be color-coded and then compared to those with a BMI less than 25. An individual could see where their tweets appear on the map, the foods and behaviors associated with this location, and how they compare to others with similar or different characteristics. This could be a powerful tool for self-realization, identifying like-minded individuals to provide social or group support, or finding dissimilar groups who represent a goal likeness and exhibit specific behaviors around which goal setting could occur. Inclusion of additional parameters could also inform the design of tailored messages sent to the participant as part of specific interventions targeting at-risk individuals or groups [23,24].

The novel use of Twitter and subsequent data visualization can provide individuals and groups with what consumers of health information have come to expect from mobile health software and health information delivery—immediate, personalized, and participatory information. Twitter was chosen, in part, because it allows tweets from third-party applications (eg, from a range of mobile phone platforms) to be accepted and displayed. The personalized nature of our platform has the capacity to deliver specific and tailored interventions to individuals based on a number of complex and interrelated characteristics. The Twitter format allows for easy 2-way communication, facilitating informal interactions between individuals (eg, clinician-patient or participant-participant). The significant amounts of real-time data collected through this system could also be used to design disease prevention interventions based on predictive algorithms. The existing social media infrastructure provided by Twitter could also be capitalized upon if and when more social and group interactions are incorporated into the process. The use of online social forums for disease prevention and health promotion activities has demonstrated utility in supporting health behavior change [25]. However, creating an appropriate environment for health behavior change through Twitter must be considered. Research suggests that as a delivery platform, the success of Twitter as a health behavior tool may be enhanced with the inclusion of tailored messaging that directly targets the individual [26,27]. We are currently incorporating this facet into our iPhone application.

A secondary objective of this study was to evaluate the acceptability of this method to collect dietary data. Participants indicated they would like to see their own data presented in a graph or similar format. Participants also wanted the ability to track their intake and reasons for eating over a longer duration to discern trends. Many users indicated that the most problematic part of the overall experience was remembering to use the study-provided list of hashtags. In this study, misspellings accounted for a small but important percentage of hashtags. This challenge has already been resolved through a feature on our iPhone application that provides participants with a list to choose from, thus eliminating need for manual entry of data.

### Limitations

A technical limitation of our study was fragmentation in canonical maps (wherein countries of a similar color were scattered across the map instead of grouped together as a solitary country). This was a software limitation caused by our limited sample size (several thousand rather than several million observations, which the GMap software is capable of), and can be resolved with a larger number of observations.

Another potential limitation was the possibility that our participants had previous experience (and comfort) using Twitter. College-age individuals use Twitter more frequently than the general public (25% vs 8% in the US population). [13]. However, 25% is still relatively low; therefore, training was provided to all participants prior to data collection to insure everyone was equally capable and comfortable collecting and posting information using Twitter.

To increase usability, we have built our own custom iOS application to simplify the reporting process. Using our application, an individual has only to click once to input the appropriate hashtag. We plan to test the application to establish the evidence necessary to optimize design and demonstrate generalizability. As smartphone ownership and social media use is projected to continue to increase, we do not anticipate that participants outside college age will have major challenges using our application or approach. However, additional studies are needed to verify this.

### Conclusions

Our findings suggest that the use of Twitter combined with a novel method of data visualization can provide a tool for identifying patterns in and relationships between food and reasons for eating, as well as additional parameters of interest (eg, temporal trends). Future research will establish the efficacy of this method in diverse populations, building the evidence base for mobile-inspired approaches to diet- and health-related behavior change for preventing obesity and optimizing population health.

## Multimedia Appendix 1

Survey.

## Multimedia Appendix 2

Details of data visualization.

## Abbreviations

BMI: body mass index
mHealth: mobile health
