# Supplementary material for: Collection and Visualization of Dietary Behavior and Reasons for Eating Using Twitter
Source: J Med Internet Res. 2013 Jun 24;15(6):e125. doi: 10.2196/jmir.2613 (PMC3713881; doi:10.2196/jmir.2613)
Supplement: Supplementary file 1 [file jmir_v15i6e125_app1.pdf]

ViBE exit survey

Exit this survey

***1. How are you accessing twitter?**

- [ ] Mobile phone
- [ ] Desktop
- [ ] Both

If you used both, which did you prefer?

***2. What application are you using to access twitter? (i.e. plume, ubertwitter, tweetcaster)**

***3. Tell us about any problems you had reporting your food intake:**

***4. Please tell us whether the foods you reported represent your "typical" food habits. If this is not your typical diet, please tell us why not.**

***5. Were you able to represent your food intake accurately with the given tags? Were there any additional hash tags you recommend we consider using in the future?**

***6. Was the training that you received adequate to prepare you to report your food intake via twitter? Please tell us what we could do to improve training.**

7. Rate the usability of the applications on a 1-5 scale (1=not difficult, 5=difficult):

	1	2	3	4	5
Twitter	○	○	○	○	○
Mobile phone	○	○	○	○	○

Why did you choose this rating?

***8. What kind of report or feedback would you most like at the completion of the study? (i.e. graphs, write-up)**

***9. Please give us suggestions of how we can improve the overall experience of the study:**
