# Supplementary material for: Collection and Visualization of Dietary Behavior and Reasons for Eating Using Twitter
Source: J Med Internet Res. 2013 Jun 24;15(6):e125. doi: 10.2196/jmir.2613 (PMC3713881; doi:10.2196/jmir.2613)
Supplement: Supplementary file 2 [file jmir_v15i6e125_app2.pdf]

Multimedia Appendix 1: Details of Data Visualization

GMap (1) generates a layout of the graph and then (2) clusters vertices based upon similarity in order to (3) produce a map of "countries". Each country corresponds to a cluster, where adjacent countries indicate strongly related clusters (FIGURE 3A and 4A). In step (1), the vertices of the graphs are assigned vertices *xy*-coordinates in an attempt to best obtain edge lengths inversely proportional to their weight. This is done using either a scalable force-directed [1] or a multidimensional scaling (MDS) [2] layout algorithm, corresponding to running the `fdp` and `neato` GraphViz layout commands, respectively. We chose `fdp` instead of `neato` to create the canonical maps. Each vertex is then labeled; but labels can become unreadable if they overlap. To solve this problem, the prism algorithm eliminates overlaps by repositioning labels [3]. In step (2), the GraphViz `cluster` command is run to group related hashtags into meaningful clusters using a modularity-based clustering algorithm [4]. For instance, all hash tags pertaining to grains may become a cluster. Finally, in step (3), the GraphViz `gvmap` command, described next, generates a geographic map representation of the data.

The `gvmap` command creates a Voronoi diagram [5], using the boundaries of the labels so that Voronoi cells can be grouped into the clusters forming "countries". This leads to the formation of "continents" and "islands" bounded by a surrounding ocean leading to natural-looking borders. To promote readability, each country is assigned a unique color. Ideally, adjacent countries should not be colored too similarly. Given that this is an NP-hard problem [6], a greedy heuristic is used.

References:

1. Fruchterman TMJ, Reingold EM,. Graph drawing by force-directed placement . Software: Practice and Experience 1991; 21(11):1129 <last_page> 1164.

2. Kruskal J, Wish M. ,Multidimensional scaling : Beverley Hills ; Sage Publications; 1978.

3. Gansner ER, Hu Y,. Efficient node overlap removal using a proximity stress model . IN 16TH SYMP ON GRAPH DRAWING (GD 2008:206; 206-217; -217.

4. Newman M,. Modularity and community structure in networks. . Proceedings of the National Academy of Sciences of the United States of America 2006; 103(23):8577-82.

5. Aurenhammer F,. Voronoi diagrams---a survey of a fundamental geometric data structure . ACM Computing Surveys 1991; 23(3):345 <last_page> 405.

6. Hu Y, Kobourov S, Veeramoni S,. On maximum differential graph coloring . IN 18TH SYMP ON GRAPH DRAWING (GD 2010.
